# Development of an eHealth Intervention Prototype to Prevent Health Risk Behaviors Among Hispanic Adolescents: A User-Centered Formative Study

**DOI:** 10.3390/ijerph21121613

**Published:** 2024-12-01

**Authors:** Yannine Estrada, Alyssa Lozano, Padideh Lovan, Devina J. Boga, Lara Martinuzzi, Jennifer Chavez, Maria I. Tapia, Guillermo Prado, Victoria Behar-Zusman

**Affiliations:** 1 School of Nursing and Health Studies, University of Miami, Coral Gables, FL 33146, USA; 2 Sylvester Comprehensive Cancer Center, University of Miami, Miami, FL 33136, USA; phl31@med.miami.edu; 3 Department of Public Health Sciences, University of Miami, Miami, FL 33136, USA

**Keywords:** Hispanic, adolescents, eHealth, risk behaviors, drug use, prevention

## Abstract

Health risk behaviors continue to disproportionately affect Hispanic youth. Despite the existence of successful family and school-based interventions, there is a need for developing and testing individually-based preventive interventions that are easily accessed and widely disseminated. Therefore, this study aimed to develop a prototype (proof of concept) for an individual-level mobile application (app), informed by Hispanic parents and adolescents, to prevent/reduce drug use and sexual risk behaviors among Hispanic youth. An iterative user-centered approach was used to inform the development of the app prototype via focus groups with 66 participants (*n* = 46 adolescents, *n* = 20 parents). A coding team analyzed data from the focus groups and identified major themes. The coding team summarized interview data into sub-categories that yielded five intervention modules for Hispanic adolescents, three more than originally proposed (i.e., drug use and sexual risk behaviors): (1) effective communication, (2) depression, (3) sexual health, (4) drug use, and (5) mindfulness. A mobile application for health risk behaviors can be used as an additional preventive tool to decrease the existing behavioral health disparities among Hispanic youth. Incorporating a user-centered approach to inform development is important for including the needs and voices of this population.

## 1. Introduction

A robust research literature on adolescent populations has shown associations between drug use and sexual risk-taking [1,2,3]. In 2023, the CDC reported that 48% of high school students did not use a condom the last time they had sex [4] and, of these, 19% reported alcohol or drug use before their last sexual encounter [5]. Subgroup data show that Hispanic adolescents are more likely to engage in drug use and sexual risk behaviors compared to other adolescents in the US [6] and that these risk behaviors tend to cluster together and have shared risk factors [7], for example, immigration-related experiences and acculturation. Given the co-morbidity of health risk behaviors in youth and the risk they pose for negative health-related outcomes later on in life, it is important to jointly target the prevention of behaviors that are highly associated, such as drug use and sexual risk behaviors [7,8,9,10,11], and account for cultural variables that impact these behaviors among Hispanic youth.

Although evidence-based prevention interventions for drug use and sexual risk behaviors exist, face-to-face interventions are limited by costs, scheduling logistics, fidelity to the intervention model, and low implementation and dissemination [12]. Technology-based interventions, often referred to as eHealth interventions [13], ameliorate some barriers that characterize face-to-face interventions. Advantages of computer eHealth preventive interventions, such as those offered by a mobile application (app) modality, include lower delivery costs, increased intervention fidelity, and greater dissemination possibility, among others [14]. eHealth interventions delivered via apps may be especially appealing to youth [15,16,17] and particularly suitable for sensitive topics, such as drug use and sexual risk behaviors, given that access can be convenient and private and learning can be personalized and self-paced [18,19]. eHealth technologies can support changes in health behavior and aid prevention and can save cost and time [20].

Despite extant health disparities and unmet service needs, eHealth interventions that target co-morbid risk behaviors for Hispanic youth are limited. Few eHealth individual-level adolescent-only prevention interventions have been developed and tested to target adolescent drug use and sexual risk behaviors [21,22]; even fewer are available that specifically target Hispanic youth, an unfortunate situation given the perennial health disparities they experience [23]. Moreover, the limited number of behavioral preventive interventions that do exist often remain at the efficacy stage without ever moving toward effectiveness or implementation [24]; therefore, it is necessary to develop preventive interventions that can easily be brought to scale, such as eHealth interventions [25,26]. Due to the intersecting and parallel development of risk behaviors [7,10], such as drug use and sexual risk behaviors, interventions that target one outcome should incorporate features of the other.

A review of technology-based youth- and family-focused interventions yielded 11 youth-focused randomized controlled trials [21]. Of these, none of the outcomes focused on both sexual risk behaviors and drug use [21]. In addition, a Centers for Disease Control report reveals that for all ages, the least likely taught topics in schools are how to get and use a condom [27]. For drug use interventions, the research literature demonstrates mixed results and an absence of customization for targeted populations [28].

The lack of customization is unfortunate given that culturally tailored behavioral interventions are more effective among Hispanic populations compared to non-culturally sensitive ones [29]. Culturally informed interventions should be appropriate, acceptable, relevant to the targeted group, and informed through participatory research [30]. The limited number of evidence-based school drug use prevention programs for Hispanic youth means that many do not receive prevention programming, even when it is offered [31]. Furthermore, Hispanic adolescents may not feel comfortable talking about sexual risk behaviors and drug use with their parents; moreover, face-to-face modalities may not work for some busy Hispanic families [32]. Overall, it is uncommon for prevention interventions to account for cultural variables that are highly salient for Hispanic youth, including gender roles and cultural values [33].

Few interventions have been developed utilizing a mobile application format [34] despite research showing that youth who participate in behavioral health programs prefer technological platforms over face-to-face delivery [35,36]. Research is needed to produce new age-specific and culturally tailored interventions to reduce health disparities related to co-morbid behaviors (i.e., drug use and sexual risk behaviors) among Hispanic adolescents in the U.S. [37] and engage adolescent youth in the research development process for these interventions. The magnitude of need in regard to prevention interventions greatly outstrips the capacity of systems to deliver preventive interventions, making traditional face-to-face intervention delivery insufficient [21]. Therefore, targeted strategies such as adolescent-only interventions delivered via mobile apps are needed to help prevent drug use and sexual risk behaviors. As such, this paper describes the user-centered formative process used to develop SEEK (socio-emotional efficacy and knowledge), a mobile app prototype designed with youth and parent input to prevent drug use and sexual risk behaviors. The aims of the study were to (1) develop an interactive mobile app prototype for an individual-level eHealth preventive intervention informed by Hispanic adolescents and parents and designed for Hispanic adolescents, to prevent/reduce drug use and sexual risk behaviors, and to (2) utilize an iterative approach to test and collect participant feedback on the developed app prototype.

## 2. Materials and Methods

### 2.1. Recruitment

Sixty-six Hispanic parents and adolescents (20 parents and 46 adolescents) from Miami-Dade County, FL, were enrolled in this formative study. Adolescents and parents were recruited via word-of-mouth through schools. Additionally, families from prior studies who agreed to be contacted for future studies were invited to participate. The research staff used purposive sampling such that an equal number of male and female adolescents were included in the study. Eligible families (a) were of Hispanic origin, defined by being born in a Spanish-speaking country of the Americas or self-identified as Hispanic, and (b) had an adolescent between the ages of 14–17. Eligible parents and adolescents were not required to have an adolescent or parent, respectively, to participate in the study. Adolescents were allowed to participate across different study phases. Consent and assent were obtained in-person for the first study phase and electronically following the discontinuation of in-person research due to the COVID-19 pandemic. All study procedures were reviewed and approved by the University of Miami Institutional Review Board.

### 2.2. Data Collection and Development

**Figure 1:**
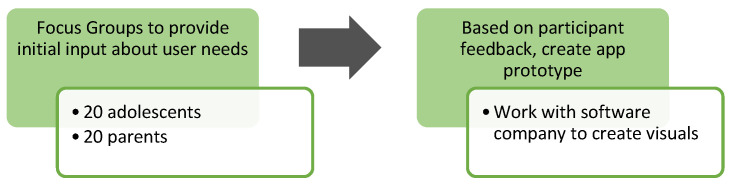
Phase 1 study design.

We used a multi-phased iterative user-centered design approach to obtain an understanding of the needs and preferences of the intervention’s end users (i.e., the adolescents). While there are multiple ways to carry out user-centered design [38], our process is informed by the literature, including an initial focus group, testing of the initial app prototype, modifications to the app prototype, and final testing [39] (see Figure 1). This information was then used to iteratively develop intervention content and measure usability. Throughout the development process, the research team created visual mockups, known as paper prototypes (from here forward app prototypes).

### 2.3. Phase 1: Initial Focus Groups and Creation of the App Prototype

The first stage of our user-centered design was to conduct focus groups to inform intervention content and technical development (Figure 1).

Focus groups are used to obtain participants’ perspectives on a particular topic. In total, six focus groups were conducted with 40 participants—2 parent-only focus groups (10 parents in each) and 4 single-gender adolescent-only focus groups (5 adolescents in each). The parent focus groups included guiding questions such as: “What type of information do you think is important for your adolescent to receive in regard to preventing drug use and sexual risk behaviors?” “Is there any type of information that you would not want your child to receive?” “What are your hopes/fears for how an intervention like this could influence your teen?” and “What are your thoughts about your adolescent receiving an online intervention to prevent health risk behaviors?” During the adolescent focus groups, adolescents were first primed for discussion about a sensitive topic by showing them video clips of an evidence-based eHealth family intervention [40] for Hispanic adolescent drug use and sexual risk behaviors. The adolescents were then asked to critique the intervention components and offer their ideas for the improvement in the intervention. The purpose of these questions was to break the ice and encourage youth to discuss these topics. This was followed by guiding questions including “What type of information would you like to receive from a mobile app health intervention that targets drug use and sexual risk behaviors?” and “What are some cultural values among Hispanics about how to act?” And relatedly, “How do you think your culture affects the decisions you make about drug use and/or sexual risk taking?” Both parent and adolescent focus groups were conducted with a bilingual facilitator (delivered in Spanish for the parents and in English for the adolescents) and lasted one hour. All focus groups were audio recorded and later transcribed in their original language. Parents and adolescents were each compensated USD 60 for their participation in the focus groups.

### 2.4. Phase 2: Testing of the App Prototype; Modification and Final Testing

**Figure 2:**
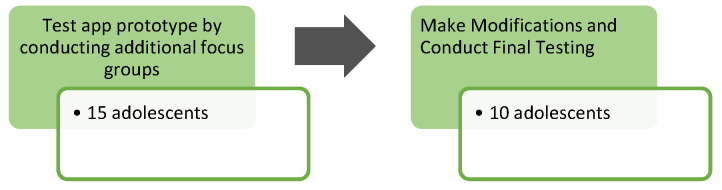
Phase 2 study design.

During these focus groups, adolescents were shown slides of the intervention and asked to interact with them by pointing to the features/links that they might select (Figure 2).

As participants interacted with the app prototype, they were presented with the next ‘screen’ and asked for feedback as they made their selections to better understand their navigation of the application, identify problems in the design process, and develop suggestions for improvement.

At the end of the focus groups, the adolescents completed the USE Questionnaire, a 30-item measure assessing the app prototype’s usefulness and ease of use [41] that includes questions such as “I am satisfied with it” and “It is useful” rated on a scale from 1 = strongly disagree to 7 = strongly agree. Participants were then asked the following questions: “What did you like most/least about the mock app?”, “How could it be improved?” and “How likely are you to use an app like this and why?”. Each of the focus groups was an hour long, video recorded, and transcribed. Adolescents were compensated USD 60 for their participation.

Lastly, after the identified concerns from the above session, the app prototype was updated, and the final two groups of adolescents (11 adolescents total) were asked to review the prototype. Using the same procedures as above, adolescents interacted with the app prototype and then completed the USE Questionnaire. Surface level modifications were made such as placement of buttons, phrasing of prompts, and organization of information. Each adolescent was compensated USD 60 for their participation.

### 2.5. Qualitative Analysis

A general inductive approach was used to establish clear links between the research objectives and summary findings derived from the raw participant data and condensed the data into a summary format to develop the SEEK prototype [42]. Two graduate students with training in qualitative research methods read all study transcripts in detail and created one codebook for the focus groups; the two additional members of the coding team provided input and the codebook was finalized once 100% agreement on codes was reached. Upper-level categories for organizing data were derived from the study objectives. These categories were topics/categories to be addressed, format, content, Hispanic-specific barriers, privacy, length of use, and reward system. Lower-level subcategories were derived directly from participant responses. The coding team was split into pairs of raters, each of whom coded 2–3 study transcripts in their original language (English or Spanish) using the codebook. Each member of the coding pair independently coded each transcript prior to meeting with the other member. Raters then met to discuss coding, reach a consensus on remaining discrepancies, and calculate percentage agreement as an indicator of inter-coder agreement; percentage agreement on independently coded data for all coding pairs was high (91%) and raters reached a full consensus on all final codes. Raters entered all final codes into Dedoose 9.0 [43], a software program used to organize and categorize qualitative data.

## 3. Results

The average adolescent age was 16 (SD = 0.77). Rating pairs applied 52 codes, 594 times across 11 focus groups, producing 454 excerpts that were relevant to the prototype’s (1) content, (2) format, and (3) privacy.

### 3.1. Phase 1 Results: Initial Focus Groups

#### 3.1.1. Content

Parents and adolescents agreed that the app prototype should include information about sexual health, the consequences of drug use, peer pressure, mental health, effective communication, parental involvement, and gender roles in Hispanic culture.

##### Sexual Health

Related to sexual health, there was a consensus among parents and adolescents that there was a need for information related to the risks associated with unprotected sex. A parent noted that the application should, “really emphasize protection if they [adolescents] are going to have intimate relationships. It’s very important to prevent any kind of disease”. Adolescents noted that one way to get this message across would be by highlighting the negative consequences of unprotected sex: “maybe like the potential outcomes of having unprotected sex, like other diseases you could get, what they could do to you, early pregnancies…all that stuff that could affect you in a negative way”. Some adolescents also stated that information about sexually transmitted infections needed to be presented graphically and visually.

Although parents and adolescents agreed on information related to contraceptives; one mother noted that Hispanics may fear that promoting condom use may be seen as encouraging sexual behavior, “Logically, among Hispanics, we sometimes have the false notion that if you give your child condoms it’s because you are giving them the liberty to go and do it. For me, no, because I am protecting my son”. Adolescents also emphasized the need for information about different methods of contraception, “other ways that, like, women can protect themselves because, you know, like, in school they only teach you, like two things. Like, it’s either condoms or birth control…it’s important that people learn other ways that they can protect themselves”. In addition to other methods, resources related to how one could obtain contraceptives would also be useful.

##### Drug Use

Adolescents were interested in information about different kinds of drugs, specifically pertaining to specific short- and long-term effects, “How much these drugs affect you or how much they can affect you, how addictive they can be.” Parents also noted that information related to how drugs could negatively affect adolescents would be important: “Same with the drugs, the effect that can cause one to have an accident, people who are disabled due to using drugs or alcohol.” Consequences of drug use outside of physical health would also be beneficial for adolescents:

Not only the health consequences, but like the laws so people can be more aware about what’s going on, like what your rights are if you get caught and what your–like how great the consequences are in case you get caught selling, ingesting, or any other use of the drug.

Other information should be specifically related to how drugs might affect Hispanics and the existing stigma associated with Hispanics’ drug use, as one adolescent said “Statistics about how these drugs affect your minority group, especially if it’s made for Hispanics and a Hispanic group, the stigma towards us, and like the stigma towards the drugs that we have and the ones that we claim”. Parents were also cognizant of the challenges associated with being Hispanic and substance use. Parents noted the normative drinking behaviors that their children are exposed to “In our Latin culture, from an early age, we’re drinking, and we do it normally. It’s part of our culture of celebrating, not only on certain dates, but in general, and that’s something we also have to expose our kids to”. Such cultural nuances would have to be addressed when discussing substance use for Hispanic adolescents.

##### Peer Pressure

Parents and adolescents agreed that in the context of discussing sexual health and drug use, material on how to resist peer pressure should be covered. Adolescents spoke about the pressures related to fitting in and how they would need guidance on how to say no in different settings:

It’s like, oh, you want to fit in, so it’s like you’re gonna go. And then once you realize, you’re like, oh, crap, what’s happening? You just don’t know how to say no at that point. You just don’t know. Like, oh, like, if you say, ‘Oh, no, I’m good’, and they’re like, ‘Come on, girl, let’s just do it’, and sometimes it’s really hard, like people don’t know how to say no or to just stop.

Parents also knew that some adolescents might do anything to fit in, one parent noted the following: “There comes a time when you’re surprised at what they’re capable of when they want to be accepted in a setting”. Given this possibility, there is a need for tools to give adolescents the strength to say no.

##### Mental Health

There was consensus that perhaps the reasons why adolescents even engaged in risky behaviors were due to underlying mental health symptomology, as one adolescent noted, “I feel like a lot of people do all those drugs and all those risky behaviors because of mental health issues that’s going on, underlying like issue that’s going on”. Given this possibility, adolescents agreed that having mental health content would be important to have within the application, “Have like maybe a mental health component of the app be like, you’re not alone, people go through this. It’s fine. You’re gonna get over it, and it’ll be a brand new day”. Parents offered insight as to how to address this topic; utilizing methods that address self-esteem and include a variety of information: “How to make it more for their self-esteem, their esteem…So we have to put the symptoms, the effects, the statistics. That type of information should be essential”. Adolescents were keenly aware of what other youth might go through and offered input on the specific mental health topics that should be discussed: “I think it should be like more like also like mental illnesses like depression or something, ‘cause a lot of people our age also go through like suicidal thoughts and depression and stuff that can also self harm them”. One example of how to address mental health-related concerns would be to simply provide positive motivational messages throughout the application, as one adolescent noted:

There’s more than one way to make sure that a person feels happy and makes them feel motivated. Telling them that they’re not alone helps a lot for people. Stuff like that. Just like a sense of motivation. A sense of motivation can help them.

“Ideas on how to communicate with us, how to approach the Hispanic parent when it comes to sex and drugs”.

##### Effective Communication

A topic area that parents and adolescents felt should be embedded throughout the application was effective communication. Adolescents noted that it was difficult to approach their parents to have conversations about sensitive subjects and that guidance on how to approach their parents would be important for the application:

Because, like, I generally don’t know how to go up to her and talk about things like that, because that—again, is awkward between both the parent and the adolescent, so, like, I feel like maybe more of, like, ways to show, like, motives to go up to your mom or dad, whoever it may be, to talk about those things. Like, I think that would be beneficial, like, for the app.

Despite the potential for awkward conversations, youth were aware that even when conversations take place, sometimes they were not effective due to a lack of understanding between parents and adolescents:

Sometimes, I feel like my mom doesn’t understand, like, where I’m trying to come across, but then I feel like sometimes, I don’t take her full viewpoint into consideration as well. So, I feel like maybe learning how to understand each other better would, like, help in talking about these things.

Therefore, tools on how to communicate with parents would be useful for adolescents, and parents mirrored that sentiment, with one parent noting “… In the application there should be ways where they give them”.

##### Gender Roles in Hispanic Culture

Participants agreed the topics would need to be addressed in the context of Hispanic culture, particularly related to the expectations of gender roles. Adolescents noted that their parents and families had ingrained rules and expectations about gender roles, particularly related to relationships. For example, one adolescent noted that

In general in Hispanic communities. There’s never like a place where you can actually talk to your parents and be like, ‘Look, like I have boyfriend, I have a girlfriend. Give me advice’. They’ll be like, ‘No. You’re just, you’re not going to have one, period’.

Both female and male adolescents agreed that these rules were associated with double standards that existed in the realm of both sexual health and substance use. For example, related to sexual activity, it was noted that “And I feel like, on the whole sex thing, I feel like if boys weren’t as pressured by older cousins or—or friends to—to have sex, then they wouldn’t pressure girls”. Similarly, it was clear that males had less restrictions and more ease when discussing sensitive topics:

I feel like it’s a lot different with boys than girls when they’re being raised, like, boys get to go out easier and, like, be at parties more and do everything more, so, it’s kind of like boys get it easier and like—I feel like with boys, they can talk more openly about, like, getting, like, let’s say, condoms or whatever. Like, even with my friends, too, it’s like—the guys can do whatever they want. Sleep over at their girlfriend’s house, like—it’s a lot different. Like, I feel like it’s looked more down upon for, like, a woman, like a girl, than a guy.

Parents stated that sometimes there were differences in how they spoke to their children; however, they were aware that those ideas may be dated and in today’s climate, there should not be a need for those differences:

Today, I don’t think there’s a difference in how you should talk to a girl or a boy, the consequences are the same…it be a girl or a boy. So basically, I think the message should be unified the same for a girl as a boy.

#### 3.1.2. Format

Both adolescents and parents provided insights on how to best format the app content; this included video testimonials, providing evidence-based research through statistics, and the importance of sustained engagement with the app. Adolescents detailed the use of short videos of past experiences, one adolescent noted the following:

I think at least, like, a couple of minute. I feel like if they were to make a video, like, with actual people sharing, like, their advice on the topic or, like, maybe just past stories or, like, something like that with somebody that has experience with, like, drugs and alcohol use. I feel like maybe the most a video should have is like a minute or two.

Parents mirrored this sentiment, noting that it would be important for testimonials to come from adolescents of the same age: “A testimonial from young kids who have experienced that and who have overcome that stage, but that they’re the same age, so that they can know that it’s not only another adult”. Another way to convey information to adolescents would be by providing statistics, one adolescent noted that “I feel like this is where the statistics could come in, because at our age we kind of think we know it all”. Parents agreed that providing concrete information is another way to convey information to youth: “I think that it has to be informative, it has to say the statistic so that it calls their attention, so they see the danger that they run”.

Participants recommended that one way to incentivize adolescents to go through all the application content could be through a point system within the app: “It can be like checkpoints. Like, ok, today you’re gonna accomplish this. And then once you do it, you like check it and then you get points”. In order to continue to retain adolescents, there could be a goal of reaching a premium level of the application, but it would still be important to have benchmarks along the way: “and I feel like another thing with the point system, we were talking about premium being your top goal, but I feel like there should be little benchmarks…They would give us a little badge every time we passed a module”.

#### 3.1.3. Privacy

There was also a focus on the importance of the app prototype being anonymous and confidential. Adolescents wanted the application to be a safe space where they could share information freely: “I also feel like this app should be a place where like you feel safe. So like maybe making every single username like anonymous”. Adolescents would not feel comfortable sharing any personal contact information, “And like anything that is personal information, like we were going back emails, so phone numbers and stuff, to me, that has to stay private and nothing that can be like disclosed to anybody else…” Despite the need for anonymity, it would still be important to monitor the information coming into the application in case there was an adolescent in danger:

I think that once it’s, like it could harm the person or the person is like attempting to harm someone else, and like that’s when it should not be private. That’s what I think, that like that–you know, sort of like…when the person kind of wants to harm someone else or themselves, that’s when it shouldn’t be private. So I think there should be monitoring, and it should be private for the most part.

### 3.2. Creation of App Prototype

**Figure 3:**
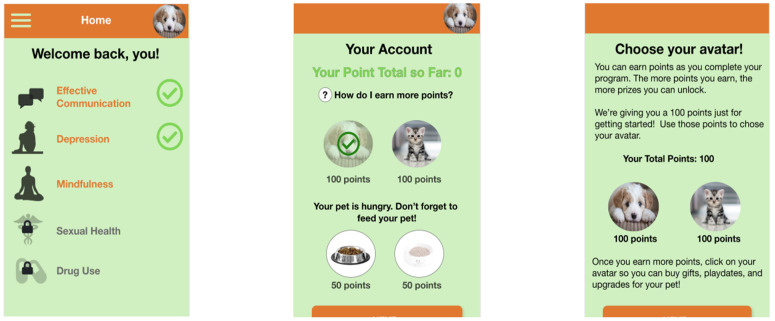
Sample app prototype.

Based on the content themes derived above, we developed prototypes for five modules (Figure 3): effective communication, depression, mindfulness, sexual health, and drug use. Because of the emphasis adolescents gave to addressing depression among youth, we made the decision to develop additional modules not initially planned at the beginning of the study (depression, mindfulness, and communication).

The drug use and sexual health behaviors modules focused on providing statistics, testimonials, and interactive exercises to help adolescents think about how to manage peer pressure and practice assertiveness skills through vignettes and ‘what if’ scenarios to help build self-efficacy in managing peer pressure. The sexual health module included interactive exercises around beliefs around sex, information on sexually transmitted infections, how to put on a condom, Hispanic gender roles, and cultural scripts around sexuality. The drug use module included interactive exercises around goals related to being drug-free, how drug use can affect adolescent’s futures, and the effects of drugs.

The communication module was included to help adolescents think about and improve their communication skills in situations where they are feeling pressured to use drugs, engage in sexual risk behaviors, or are managing feelings of depression. Information on how to communicate with parents was also included through, for example, the use of tips and tools for effective communication such as attentive listening and the use of “I” statements. Additionally, we incorporated interactive exercises to help adolescents identify what positive communication looks like and barriers to healthy communication such as properly managing feelings.

The depression module included information about symptoms; identifying and modifying cognitive distortions through interactive exercises on thoughts, feelings, and behaviors; coping strategies; and statistics on depression in adolescents. Additionally, we included problem-solving skills such as ways to overcome negative thinking and an interactive exercise to understand types of thinking that can lead to depressed mood such as overgeneralization and jumping to conclusions. We also incorporated information about how to get help and community resources available to youth, including national suicide telephone numbers.

Based on adolescent feedback regarding the importance of addressing depression, we felt it necessary to provide adolescents with additional tools for managing mental health symptoms and, therefore, created a mindfulness module for adolescents to address feelings of stress, depression, and/or anxiety. This module included an explanation of mindfulness practices, self-check-in interactive exercises, breathing exercises to increase breath awareness, mini relaxation exercises, and guided meditation.

### 3.3. Phase 2 Results: Testing of the App Prototype; Modification and Final Testing

**Figure 4:**
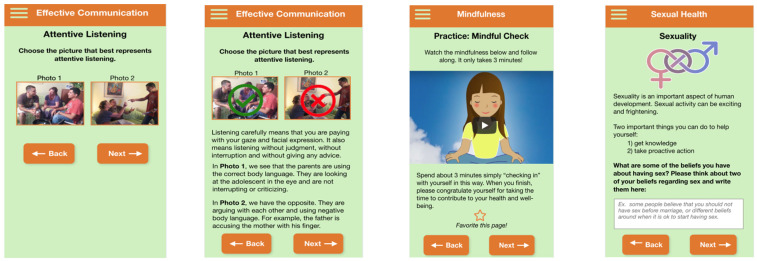
Content specific app prototype.

After the creation of the initial set of prototypes, two waves of focus groups were conducted to obtain feedback from adolescents. During the first wave, three focus groups (*n* = 15) were conducted. The full intervention modules were presented to obtain feedback on the developed prototypes and participants were asked to provide feedback on each presented “screen” and asked to “think aloud” as they made their selections. The “think aloud” method is used to understand participants’ navigation of websites, identify problems early in the design process, and develop suggestions for improvement [35]. A traditional think aloud protocol, or one in which minimal prompting is provided (i.e., no probes beyond “keep talking”) was used so as not to bias participant feedback [35]. Due to the COVID-19 pandemic, protocol modifications had to be made to conduct the focus groups online. Recommended changes were incorporated into the modules before the second wave of focus groups. For the second wave, three (*n* = 11) focus groups were conducted. During these focus groups, participants were asked specific questions about the look and feel of the prototypes, the placement of buttons, the ordering of content, and the actual content (Figure 4). The provided adolescent feedback was used to make further modifications to the prototypes. Adolescents expressed positive feedback overall.

Findings from the USE survey indicated that 80% of youth found that the prototypes would be useful, 73% felt that occasional and regular users would like it, 72% felt satisfied with it, and 80% felt that it was wonderful. Participants offered feedback regarding the look and feel of the prototypes and specific feedback regarding graphics, statistics, and content. Additionally, adolescents felt that a reinforcement system should be incorporated such that points were accumulated as modules were completed. These points could then be used to feed a pet character that would be present throughout the module.

## 4. Discussion

In this study, we developed an app prototype for an individual-level eHealth application preventive intervention designed for Hispanic adolescents that was user-centered and informed by Hispanic parents and adolescents living in South Florida. Qualitative analysis revealed three higher-order themes that were consistent across parents and adolescents to inform development of the SEEK app: content, format, and privacy. Within the content theme, parents and adolescents wanted the prototype to incorporate information on sex, the consequences of drug use, mental health, effective communication, gender roles in Hispanic culture, and peer pressure, among others. Taking this feedback into consideration, we added an additional three modules that were focused on effective communication, depression, and mindfulness. Adolescents and parents agreed on the format in which the intervention should be presented and included ideas such as having testimonials and statistics to convey information to adolescents. Moreover, adolescents identified a reward system that may incentivize adolescents to complete the intervention modules. Lastly, in the privacy theme, adolescents expressed the need for the application to be private such that it would not be linked to any personal information and recommended an anonymous login as one method through which to protect information. Despite the importance of keeping identifying information private, adolescents stressed the need to monitor the app for any serious indications of emotional or mental distress requiring professional help.

The need for individual-level and technology-based preventive interventions such as SEEK is highlighted by the continued health disparities Hispanic youth face, including drug use and sexual risk behaviors [1,2,3]. Despite these documented disparities, evidenced-based interventions have historically excluded Hispanic youth [44,45]. The SEEK intervention begins to address the gaps in available behavioral preventive interventions for Hispanic youth through its technology-based modality that can reach a broader population of Hispanic youth. In addition to being accessible to Hispanic youth, SEEK also appears to be useful and acceptable to Hispanic youth; 80% of youth found the app prototype would be useful and 73% felt that occasional and regular users would like it. Further, SEEK can potentially be a preventive intervention

The present study utilized feedback from both the parent and the adolescent to inform the development of intervention modules to target adolescent risk behaviors among Hispanic youth—an often-underrepresented group in the research literature. The inclusion of parents, even though they are not the end users of the application, ensures that the developed application is acceptable to them so that they would allow and encourage their youth to use the application. The parent perspective further supplements the youth’s perspectives; too often, intervention components are developed without consideration for the perspective of the individuals who will use them, particularly groups who are underserved such as Hispanic youth. Indeed, the importance of including youth voices in research is highlighted by seminars focused in this area sponsored by organizations such as the National Academies of Sciences, Engineering, and Medicine [46] and in reviews highlighting the need for better app developer-user collaborations [47].

The current study elevated the voice of Hispanic youth wherein they shaped the content of the intervention prototypes by expressing the need for a depression module, one that was not an initial module at the beginning of the study and was also a driver for the incorporation of the meditation and communication modules. This subsequently expands the outcomes that are addressed in the SEEK intervention and addresses the pervasive mental health disparities that impact Hispanic youth. Moreover, as drug use, sexual risk behaviors, and depressive symptoms are increasingly found to be comorbid with each other, interventions such as SEEK will fill a large gap in the intervention research literature.

The present study should be interpreted while considering the following limitations. First, all participants were from one geographic area in South Florida, which limits the generalizability and representation of Hispanic subpopulations. Second, some of the focus groups took place during the COVID-19 pandemic; this may have influenced participant feedback given the larger context of the pandemic and its known effects on mental health. Mental health continues to worsen among adolescents in the post-COVID-19 era, therefore warranting the inclusion of mental health-related content in preventive interventions for adolescents.

## 5. Conclusions

This study incorporated a user-centered approach to inform the development of SEEK, an individual-level eHealth application app-based preventive intervention for the prevention of drug use, sexual risk behaviors, and depressive symptoms for Hispanic youth in South Florida. We developed five intervention modules for Hispanic adolescents, three more than originally proposed (i.e., drug use and sexual risk behaviors): (1) effective communication, (2) depression, (3) sexual health, (4) drug use, and (5) mindfulness. The next steps include using the created prototypes as a guide to develop a fully functional app to be tested with Hispanic parents and adolescents via a pilot randomized trial. Mobile applications can begin to ameliorate health disparities related to drug use, sexual risk behaviors, and depressive symptom outcomes among Hispanic youth.

## Data Availability

Data for this study are unavailable due to privacy restrictions.
